# Optimization of colonoscope bedside precleaning protocol: an orthogonal experimental study

**DOI:** 10.3389/fcimb.2026.1704851

**Published:** 2026-05-28

**Authors:** Bingru Li, Jinpeng Jiang, Yingying Wang, Qihan Wu, Yuan Sheng, Ningning Li, Mengdan Ye, Wen Li, Wei Cai

**Affiliations:** 1 Department of Digestive Endoscopy Center, Nanjing Drum Tower Hospital, Affiliated Hospital of Medical School, Nanjing University, Nanjing, China; 2 Department of Infection Management Office, Nanjing Drum Tower Hospital, Affiliated Hospital of Medical School, Nanjing University, Nanjing, China

**Keywords:** bedside precleaning, endoscope reprocessing colonoscope, decontamination, experimental, orthogonal

## Abstract

**Objective:**

This study utilized an orthogonal experimental design to optimize the bedside precleaning protocol for colonoscopes. It evaluated the correlation between adenosine triphosphate (ATP) bioluminescence testing and microbial culture results, thereby establishing an evidence-based foundation for quality control in endoscopy.

**Methods:**

Four factors affecting colonoscope bedside precleaning were selected, each with three levels, and arranged into nine protocols using an L9(3^4^) orthogonal design. One hundred eighty colonoscopes were assigned to nine groups (n = 20) for different bedside precleaning protocols after the procedure. ATP levels (RLU) and microbial counts (CFU/mL) were measured after bedside precleaning, cleaning, and disinfection. Spearman correlation analysis was performed using GraphPad Prism 10.1.2, and cost-effectiveness was assessed.

**Results:**

Factors A (types of detergents) and C (suction duration) demonstrated statistically significant effects on post-precleaning ATP values and microbial counts (P = 0.035 and P = 0.048, respectively), with alkaline solution (A_3_) and 20-second suction (C_2_) showing optimal efficacy. No factors significantly influenced postcleaning ATP levels or microbial counts (P>0.05).

**Conclusions:**

The optimized colonoscope bedside precleaning protocol for reducing biopsy channel contamination includes: alkaline solution (A_3_), solution replacement every four hours (B_2_), 20s suction (C_2_), and a clinically appropriate delay (≤3h). ATP testing and microbial culture exhibit high consistency in assessing biopsy channel cleanliness during cleaning, which can serve as a useful screening tool for immediate process intervention during endoscope cleaning. However, its utility post-HLD is limited.

## 1 Introduction

In gastrointestinal endoscopy, the continuous increase in procedure volume and turnover rates has made endoscopy-associated infections a significant global concern for healthcare-associated infections (Calderwood et al., 2018; Wang et al., 2022). Current international endoscope reprocessing protocols consist of bedside precleaning, leakage testing, manual cleaning, rinsing, high-level disinfection, final rinsing, drying, and storage (Beilenhoff et al., 2018; Day et al., 2021; British Society of Gastroenterology, 2020; Liu et al., 2017). However, even with strict adherence to guidelines, suboptimal reprocessing remains a prevalent issue. A nationwide multicenter study revealed a reprocessing non-compliance rate of up to 36.81% (Casini et al., 2023).

Evidence demonstrates that immediate cleaning after procedures improves reprocessing efficacy (Loyola et al., 2020; Shang et al., 2023). However, immediate cleaning is often impractical in clinical practice with high workflow density and resource limitations. As an immediate and targeted procedure, bedside precleaning is crucial for optimizing reprocessing (Meeusen and McLean, 2023).

Bedside precleaning is defined as the immediate cleaning of an endoscope performed at the bedside of the procedure following its withdrawal from the patient. It is conducted before disconnecting the device from the light source and video processor, and entails wiping the external surface and aspirating detergent through all internal channels (Eberhardt et al., 2024). This critical step aims to reduce the adherence of organic debris and facilitate subsequent cleaning. Although research on precleaning has increased recently, available studies remain fragmented and lack systematic evaluation. While existing studies have examined factors such as types of precleaning detergents (Gonzalez et al., 2019), patented precleaning devices (Wang et al., 2023), and delayed cleaning intervals(Chen et al., 2024), neither definitive conclusions nor optimized protocols have been established. Furthermore, current guideline recommendations on precleaning remain vague and lack clinical specificity (Calderwood et al., 2018;Beilenhoff et al., 2008; Day et al., 2021; British Society of Gastroenterology, 2020; Liu et al., 2017).

Therefore, this study focuses on optimizing endoscope precleaning. Through a comprehensive literature review and preliminary clinical investigations, we identified key factors affecting precleaning efficacy. We aim to establish an optimized colonoscope precleaning protocol based on biopsy channel contamination assessment using an orthogonal experimental design. Additionally, by examining the correlation between relative light units (RLU) and colony-forming units (CFU/mL) in biopsy channel samples, we evaluate the clinical utility of ATP bioluminescence as a rapid quality monitoring tool in endoscope reprocessing.

## 2 Materials and methods

### 2.1 General information

This single-center interventional study was conducted at a tertiary endoscopy center in Jiangsu Province (annual procedure volume: 110,000 GI endoscopies) between January and March 2025. The investigation focused on colonoscopes (Olympus CF-H290l; working length: 1330 mm; biopsy channel diameter: 3.2 mm).

### 2.2 Methods

This was an orthogonal experimental study conducted to optimize the colonoscope bedside precleaning protocol. Based on a preliminary literature review and clinical bedside precleaning workflow analysis, four critical factors with three test levels each were identified for optimization. The study employed an L9(3^4^) orthogonal array design, with details presented in **Table 1**.

**Table 1:** Orthogonal array design for pre-cleaning protocols.

Protocol	A	B	C	D
1(A_1_B_1_C_1_D_1_)	water	each use	10s	0min
2(A_1_B_2_C_2_D_2_)	water	every 2 hours	20s	1h
3(A_1_B_3_C_3_D_3_)	water	every 4 hours	30s	3h
4(A_2_B_1_C_2_D_3_)	enzymatic detergent	each use	20s	3h
5(A_2_B_2_C_3_D_1_)	enzymatic detergent	every 2 hours	30s	0min
6(A_2_B_3_C_1_D_2_)	enzymatic detergent	every 4 hours	10s	1h
7(A_3_B_1_C_3_D_2_)	alkaline detergent	each use	30s	1h
8(A_3_B_2_C_1_D_3_)	alkaline detergent	every 2 hours	10s	3h
9(A_3_B_3_C_2_D_1_)	alkaline detergent	every 4 hours	20s	0min

Inclusion criteria were as follows: (a) colonoscopes (Olympus CF-H290I, effective length 1330 mm, biopsy channel diameter 3.2 mm, Japan) used only for routine diagnostic examination without any additional therapeutic procedure; (b) patients undergoing colonoscopy were aged 18 to 60 years. Exclusion criteria were as follows: (a) colonoscopes used in patients with documented drug-resistant bacterial infection requiring specialized disinfection; (b) colonoscopes found during reprocessing to have instrument damage such as water leakage. All consecutive colonoscopy procedures that met these criteria during the study period were enrolled.

A dedicated nurse performed all bedside precleaning procedures, which were conducted strictly in accordance with the orthogonal design protocols. Suction procedures were maintained at a pressure of 0.04 MPa, with researchers timing the intervals using stopwatches and issuing “start” and “stop” commands. The nurse then executed the corresponding suction operations according to these instructions. After completion of precleaning, the waterproof cap was secured, and the colonoscope was placed in a sealed transport container for delivery to the cleaning and disinfection room. During delayed processing intervals, the colonoscope was stored in a decontamination area maintained at 22–24 °C with 70–80% humidity under sealed conditions(Chen et al., 2024). Subsequent reprocessing steps strictly followed established guidelines: leakage testing; manual cleaning (3 min); rinsing (2 min); high-level disinfection (5 min); final rinsing (2 min)(Liu et al., 2017).

A total of 180 colonoscopes were randomly allocated to nine orthogonal protocol groups (20 per group) using a computer-generated random permutation procedure. Specifically, a random number was generated for each colonoscope using SPSS version 26.0 with a fixed random seed of 20010304. The random numbers were then sorted in ascending order, and the sorted list was consecutively partitioned into nine blocks of 20 numbers each, corresponding to protocols 1 to 9, respectively. The allocation was then mapped back to the original enrollment order, thereby producing a random assignment sequence. The dedicated nurse performed bedside precleaning strictly according to this sequence. Outcome assessors, specifically the personnel responsible for ATP bioluminescence testing and microbial culture, were blinded to group allocation. All samples were relabeled with numeric codes before being handed over to the testing laboratory, and the analyst had no knowledge of which protocol the sample originated from.

### 2.3 Observation indicators

#### 2.3.1 ATP measurement

According to the Ruhof ATP Complete^®^ Contamination Monitoring System operating manual, sampling swabs were equilibrated at room temperature for 15 minutes before use. For biopsy channel sampling, the foam-tipped swab was inserted through the biopsy port until the tip emerged from the distal end, where the protruding foam tip was excised using sterile scissors before returning the swab to its test tube. The ATP detector was preheated for 60 seconds. Afterward, the swab’s upper segment was snapped and repeatedly compressed to ensure reagent flow to the tube base, followed by gentle shaking for 3 seconds before insertion into the Ruhof handheld luminometer. Press the “OK” button, wait for 15 seconds, and record the ATP value. Results were interpreted according to the manufacturer’s instructions: a value of ≤45 RLU was considered passing, while a value of>45 RLU was considered failing.

#### 2.3.2 Colony counts

Microbiological culturing was conducted in strict compliance with the GB15982–2012 Standards for Hospital Disinfection (General Administration of Quality Supervision et al., 2012). Using a sterile syringe, 50 mL of broth was aspirated and injected into the biopsy channel, followed by complete collection of the total effluent volume. Sampling was performed at three critical stages: after bedside precleaning, after manual cleaning, and after high-level disinfection. Each sample was vigorously homogenized using a vortex mixer, and 1 mL aliquots were plated in duplicate onto nutrient agar plates. Bacterial colonization was quantified by counting colony-forming units (CFU) after 48 of incubation at 37 °C.

### 2.4 Cost analysis

The study’s cost assessment focused primarily on variable costs associated with (1) bedside precleaning Types of detergents (Factor A), (2) solution replacement frequency (Factor B), and (3) suction duration (Factor C), while fixed costs (labor, equipment depreciation, utilities) were excluded due to allocation complexities. Specific consumable costs included an enzyme detergent (4L, $208 USD) and an alkaline detergent (5L, $264 USD). The analysis calculated variable bedside precleaning costs per colonoscope across all nine experimental combinations.

### 2.5 Sample size determination

The sample size calculation was based on an established orthogonal experimental design methodology (Sun et al., 2021), where n = 20 replicates per group have been demonstrated to yield statistically robust results. Accordingly, this study ultimately enrolled 180 post-procedure colonoscope samples, which were allocated across nine experimental groups (20 samples per group) to ensure balanced group comparisons.

### 2.6 Statistical analysis

Data management was performed using Excel 2021, with statistical analyzes conducted in SPSS 25.0. Continuous variables were expressed as mean ± standard deviation (x̄ ± s) and compared using one-way ANOVA for multiple group comparisons. Categorical data were presented as frequencies (%), with between-group comparisons of compliance rates after cleaning and disinfection assessed using non-parametric Mann-Whitney U tests. Orthogonal range analysis and ANOVA were used to evaluate the efficacy of bedside precleaning and cleaning across experimental groups. Spearman’s rank correlation analysis was performed between ATP bioluminescence values (RLU) and microbial culture results (CFU/mL) using GraphPad Prism 10.1.2 (GraphPad Software, San Diego, CA), with corresponding correlation scatter plots generated. All statistical tests were two-tailed, with *P ≤* 0.05 considered statistically significant.

## 3 Results

### 3.1 Contamination status during colonoscope reprocessing

Log-transformed ATP values and microbial counts were analyzed before the disinfection process. Results demonstrated no statistically significant differences in precleaning ATP values among the nine groups (*F = 1.195, P = 0.305*), indicating comparable baseline conditions for evaluating colonoscope precleaning efficacy across all experimental configurations. Both bedside precleaning and manual cleaning achieved a 1-log reduction in ATP values, while manual cleaning alone reduced microbial counts by 1 to 2 logs. ANOVA revealed significant post-bedside precleaning differences for both ATP (*F = 10.240, P< 0.001)* and microbial counts *(F = 9.971, P<* 0.001). However, post-cleaning results showed no intergroup variation in ATP levels (*F = 0.570, P = 0.802*) or microbial counts (*F = 0.645, P = 0.739*). All groups achieved 100% cleaning compliance, with disinfection pass rates of> 90%. Non-parametric tests confirmed equivalent post-disinfection outcomes across groups for both ATP (*H = 5.449, P = 0.709)* and microbial counts *(H = 1.770, P = 0.987*). Detailed results are presented in **Table 2**.

**Table 2:** Contamination status during colonoscope reprocessing. .

Protocol	Quantity						Cleaning efficacy (%)	Disinfection efficacy(%)
		Post-use	Post precleaning		Post manual cleaning			
		RLU#	RLU#	CFU/ml#	RLU#	CFU/ml#		
1(A_1_B_1_C_1_D_1_)	20	3.70±0.20	2.53±0.23	4.42±0.28	0.91±0.17	2.33±0.70	100%	90%
2(A_1_B_2_C_2_D_2_)	20	3.60±0.21	2.40±0.17	4.32±0.32	0.82±0.33	2.56±0.49	100%	90%
3(A_1_B_3_C_3_D_3_)	20	3.59±0.20	2.32±0.28	4.25±0.26	0.83±0.32	2.59±0.45	100%	100%
4(A_2_B_1_C_2_D_3_)	20	3.59±0.21	2.15±0.13	3.97±0.18	0.92±0.17	2.31±0.74	100%	100%
5(A_2_B_2_C_3_D_1_)	20	3.54±0.21	2.11±0.15	3.96±0.23	0.92±0.16	2.28±0.76	100%	95%
6(A_2_B_3_C_1_D_2_)	20	3.56±0.21	2.28±0.22	4.24±0.29	0.89±0.24	2.30±0.74	100%	95%
7(A_3_B_1_C_3_D_2_)	20	3.57±0.16	2.10±0.20	3.97±0.20	0.84±0.29	2.44±0.61	100%	100%
8(A_3_B_2_C_1_D_3_)	20	3.55±0.15	2.25±0.23	4.18±0.29	0.79±0.40	2.43±0.58	100%	100%
9(A_3_B_3_C_2_D_1_)	20	3.56±0.15	2.12±0.22	3.89±0.32	0.85±0.27	2.47±0.55	100%	95%

#All values are presented as mean ± SD (log₁₀-transformed). RLU, relative light units; CFU, colony-forming units.

### 3.2 Range analysis of ATP values and microbial counts in colonoscopes

According to the principles of orthogonal experimental analysis, the range (R = maximum value - minimum value) reflects the magnitude of each factor’s influence on experimental outcomes, with larger R values indicating greater importance of the corresponding factor. For both post-precleaning ATP values and microbial counts, the significance ranking of factors was R_A_ > R_C_ > R_D_ > R_B_, indicating the following order of influence: Types of detergents > suction duration > precleaning-to-cleaning interval > solution replacement frequency. The K-value represents the mean effect of different levels within a factor, with its magnitude determining the optimal level for each factor. Using post-precleaning ATP levels from the biopsy channel as an example, K_1_ > K_2_ > K_3_ demonstrates that among different cleaning agents under factor A, the efficacy ranking was: alkaline detergent > enzyme detergent > water. Detailed results are presented in **Table 3**.

**Table 3:** Range analysis.

Indicator		A	B	C	D
Post precleaning RLU	K1	2.417	2.261	2.347	2.254
	K2	2.180	2.247	2.222	2.259
	K3	2.151	2.240	2.178	2.234
	R	0.266	0.021	0.169	0.025
	Factor	ACDB			
	Priority	A_3_C_3_D_3_B_3_			
Post precleaning cfu/ml	K1	4.332	4.122	4.263	4.093
	K2	4.059	4.137	4.062	4.178
	K3	3.999	4.130	4.064	4.118
	R	0.333	0.015	0.202	0.085
	Factor	ACDB			
	Priority	A_3_C_2_D_1_B_1_			
Post manual cleaning RLU	K1	0.855	0.891	0.848	0.893
	K2	0.909	0.829	0.865	0.851
	K3	0.815	0.858	0.866	0.834
	R	0.094	0.062	0.018	0.059
	Factor	ABDC			
	Priority	A_3_B_2_D_3_C_1_			
Post manual cleaning cfu/ml	K1	2.494	2.360	2.333	2.363
	K2	2.299	2.403	2.448	2.435
	K3	2.425	2.455	2.437	2.421
	R	0.196	0.095	0.115	0.072
	Factor	ACBD			
	Priority	A_2_C_1_B_1_D_1_			

### 3.3 ANOVA of colonoscope ATP values and microbial counts

Variance analysis was performed to determine the statistical significance of each factor’s impact on ATP levels and microbial counts after bedside precleaning and cleaning. Larger F-values indicate greater factor influence. For post-bedside precleaning results, both Factor A (types of detergents) and Factor C (suction duration) significantly affected ATP values (*P* < 0.001) and microbial counts (*P = 0.035 and P = 0.048*, respectively). In contrast, post-cleaning analysis revealed no significant effects from any of the factors (Factor A: solution type, Factor B: replacement frequency, Factor C: suction duration, Factor D: bedside precleaning-cleaning interval) on either ATP levels or microbial counts (*P > 0.05*). Detailed results are presented in **Table 4**.

**Table 4:** Analysis of variance.

Indicator	Factors	SS	DF	MS	F	P
Post precleaning RLU	A	1.916	2	0.958	22.403	0.000
	B	0.015	2	0.008	0.178	0.837
	C	0.293	2	0.146	3.424	0.035
	D	0.004	2	0.002	0.044	0.957
Post precleaning cfu/ml	A	2.653	2	1.327	18.704	0.000
	B	0.003	2	0.002	0.023	0.977
	C	0.438	2	0.219	3.086	0.048
	D	0.219	2	0.110	1.545	0.216
Post manual cleaning RLU	A	0.136	2	0.068	0.918	0.401
	B	0.062	2	0.031	0.419	0.658
	C	0.003	2	0.002	0.022	0.978
	D	0.079	2	0.040	0.533	0.588
Post manual cleaning cfu/ml	A	1.160	2	0.580	1.440	0.240
	B	0.283	2	0.141	0.351	0.705
	C	0.136	2	0.068	0.168	0.845
	D	0.180	2	0.090	0.224	0.800

### 3.4 Cost analysis

Detailed results are presented in **Table 5**.

**Table 5:** Cost-benefit analysis of precleaning protocols.

Protocol	Cost($)
1(A_1_B_1_C_1_D_1_)	0
2(A_1_B_2_C_2_D_2_)	0
3(A_1_B_3_C_3_D_3_)	0
4(A_2_B_1_C_2_D_3_)	0.08
5(A_2_B_2_C_3_D_1_)	0.12
6(A_2_B_3_C_1_D_2_)	0.04
7(A_3_B_1_C_3_D_2_)	0.16
8(A_3_B_2_C_1_D_3_)	0.05
9(A_3_B_3_C_2_D_1_)	0.11

### 3.5 Correlation between ATP and microbial load

**Figure 1:**
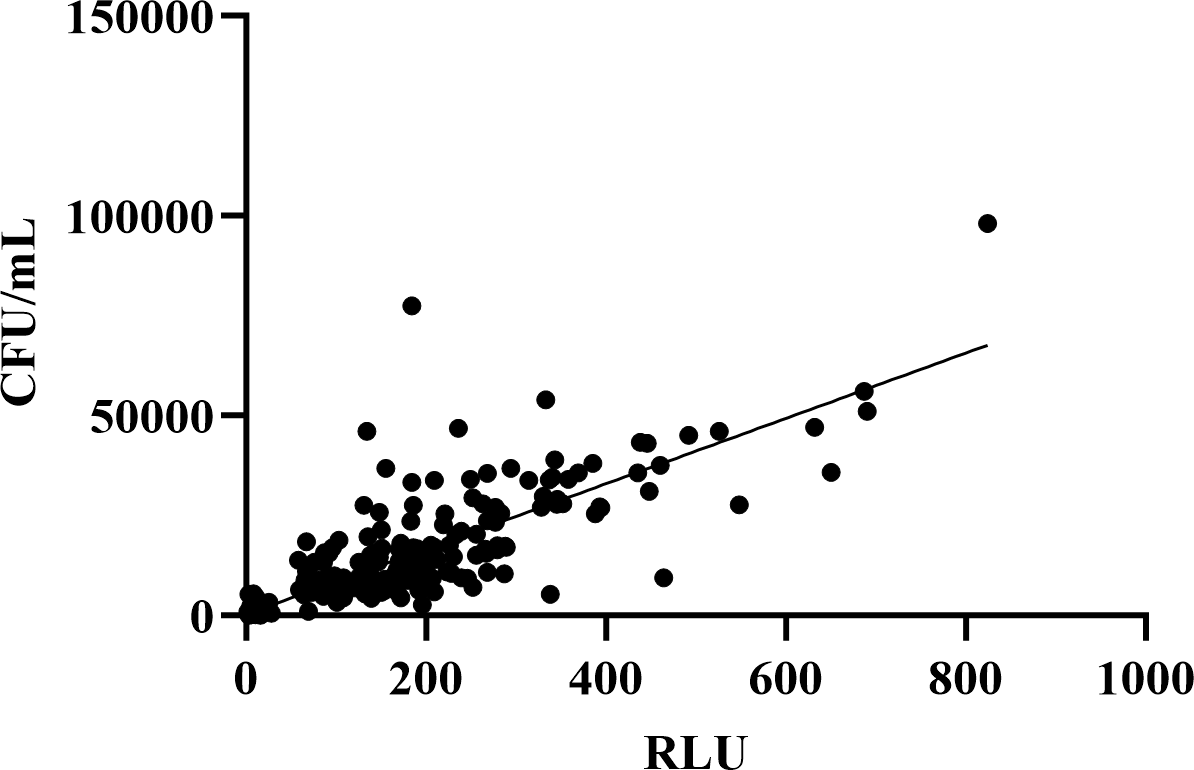
Linear regression analysis for CFU/ml and RLU during colonoscope cleaning.

**Figure 2:**
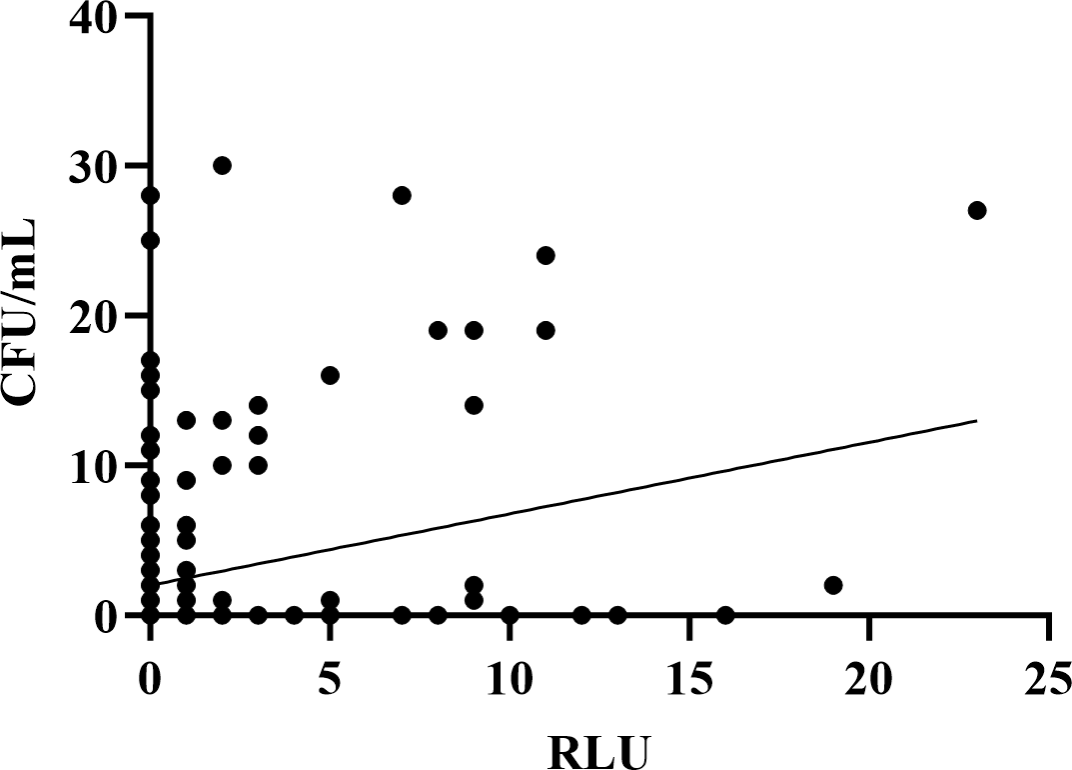
Linear regression analysis for CFU/ml and RLU after HLD of colonoscope.

A strong positive correlation was identified between RLU and CFU/mL values during the colonoscope cleaning phase (*R = 0.864, 95% CI: 0.835–0.889; P< 0.0001*), demonstrating that ATP bioluminescence accurately reflects viable microbial contamination levels at the cleaning stage. In contrast, high-level disinfection (HLD) samples exhibited only a weak correlation (*R = 0.239, 95% CI: 0.092–0.376; P = 0.0012*), indicating that ATP detection has limited utility for verifying the efficacy of high-level disinfection (HLD). Detailed results are presented in **Figures 1**, **2**.

## 4 Discussion

Current international guidelines emphasize the necessity of bedside precleaning in endoscope reprocessing, but they lack specific operational details regarding procedural steps and parameters (Calderwood et al., 2018; Beilenhoff et al., 2008; Day et al., 2021; British Society of Gastroenterology, 2020; Liu et al., 2017).

Current guidelines do not specify types of precleaning detergents, merely recommending selection according to IFU(Calderwood et al., 2018; Beilenhoff et al., 2008; Day et al., 2021; British Society of Gastroenterology, 2020; Liu et al., 2017). **Table 4** demonstrates that Factor A (types of detergents) has a significant impact on post-precleaning ATP values and bacterial colony counts (*P< 0.001*), indicating its critical role in precleaning efficacy. While enzymatic detergents containing proteases and amylases effectively degrade proteins and polysaccharides, their performance is environmentally sensitive (Gonzalez et al., 2019). Alkaline detergents induce saponification reactions with fatty acids, producing water-soluble compounds (fatty acid salts and glycerol) that maintain stable cleaning efficacy for up to 12 hours. As shown in **Table 3**, A_3_ (alkaline cleaner) demonstrated superior performance in reducing both ATP values and microbial counts, supporting its selection as the optimal type of precleaning detergent, consistent with previous findings (Chen et al., 2024a). It is important to clarify that this conclusion was obtained under specific precleaning conditions: room temperature (20–25 °C) and short contact time. The temperature condition falls within the effective range recommended by the manufacturer. Although this brief, suction-based contact differs from the several-minute soaking typically recommended for thorough cleaning, it is designed for the time-sensitive clinical procedure of bedside precleaning.

Multisociety guidelines recommend replacing detergents after each procedure or when exceeding the specified dilution ranges, although this recommendation carries a low evidence grade without clinical validation (Day et al., 2021). Studies have demonstrated that compared with traditional precleaning buckets, the use of disposable bedside precleaning kits (Patent No: ZL201920911448.7) significantly reduces ATP values in gastroscope biopsy channels. However, their higher cost poses challenges for widespread adoption in hospitals (Wang et al., 2023). Our findings indicate that Factor B (precleaning solution replacement frequency) had no significant effect on ATP values or microbial counts in the biopsy channel, either after precleaning or after manual cleaning (*P > 0.05*), suggesting that solution replacement frequency has no substantial impact on biopsy channel precleaning efficacy. Range analysis in **Table 3** further reveals that the optimal replacement frequency varies across different reprocessing stages. Considering both cost-effectiveness and practical feasibility, a 4-hour replacement interval is recommended for precleaning solutions.

European guidelines specify suction durations of 10–20 seconds (Beilenhoff et al., 2018), while others omit this parameter. Factor C (suction duration) significantly influenced cleaning outcomes (*P* = 0.035; *P* = 0.048). Wang Yang implemented process optimization using the FOCUS-PDCA methodology with one specific modification involving suction procedures: the original protocol of “continuous suction until detergents flow through the suction channel” was revised to “simultaneous suction of detergents through the biopsy channel while insufflating air through the air/water channel until clear effluent is observed(Wang et al., 2019).” However, it is essential to note that visually distinguishing between clear and turbid precleaning solutions remains challenging. Insufficient suction duration may compromise the effective removal of luminal contaminants. As shown in **Table 3**, the optimal suction duration varied. Considering both cleaning efficacy and cost-effectiveness, this study recommends a suction duration of 20 seconds as the optimal duration.

Surveys indicate that high volumes of endoscopic procedures and time constraints have led to delayed cleaning after precleaning, making it a widespread issue (Sivek et al., 2022). Guidelines suggest that when a high workload prevents immediate reprocessing, the cleaning time window may be appropriately extended, but should not exceed 3 hours (British Society of Gastroenterology, 2020). Chen employed a factorial design to demonstrate that a 3-hour delay had no significant impact on the efficacy of gastroscope reprocessing(Chen et al., 2024b). Eichel confirmed through simulated and clinical studies that the time interval between precleaning and manual cleaning did not significantly affect endoscope cleanliness, indicating that pre-cleaned endoscopes may be stored overnight before subsequent reprocessing (Eichel et al., 2021). As shown in **Table 4**, Factor D (precleaning-to-cleaning interval) had no significant effect on ATP values or microbial counts in the biopsy channel, either after precleaning or after manual cleaning (*P > 0.05*), indicating that this interval does not substantially influence biopsy channel precleaning outcomes. Range analysis in **Table 3** further reveals that the optimal time intervals vary across different reprocessing stages. Therefore, while clinical units may adjust cleaning schedules based on actual workload, the delay should not exceed the 3-hour safety threshold to maintain reprocessing standards and ensure patient safety.

Multiple studies have confirmed that ATP bioluminescence testing can serve as a valuable tool for monitoring the quality of manual endoscope cleaning. Still, it cannot replace microbial culture as the gold standard (Alfa et al., 2013; Alfa et al., 2014; Batailler et al., 2015). This study identified a strong positive correlation between RLU and CFU/mL in biopsy channel samples during the cleaning phase of colonoscopes (*r = 0.864, 95% CI: 0.835–0.889; P< 0.0001*). In this phase, endoscopic surfaces are contaminated with substantial amounts of live and dead bacteria as well as organic material, and the number of culturable live bacteria correlates strongly with the total bioburden. The strength of this correlation was significantly greater than that reported by McCafferty(*r = 0.497, 95% CI: 0.28–0.66; P< 0.0001*) (McCafferty et al., 2018). The discrepancy may be attributed to differences in sensitivity or detection principles among ATP detectors from various manufacturers.

Notably, only a weak correlation was observed between CFU/mL and RLU in samples collected after high-level disinfection (*r = 0.239, 95% CI: 0.092–0.376; P = 0.0012*), This can be attributed to the fact that high-level disinfection fundamentally alters the nature of residual contaminants. The process eliminates most culturable bacteria, leaving behind residual microorganisms that may exist in a viable but non-culturable (VBNC) state, as dead cells, or as spores. Operational steps in this study, such as delayed cleaning time and disinfectant action, create environmental stress that can induce bacteria to enter the VBNC state. While these bacteria cannot grow on culture plates, their intact cellular structures may still contain detectable ATP. Additionally, HLD cannot remove all non-cellular organic residues. CFU values reflect only a minimal number of culturable live bacteria, whereas RLU values represent the combined total of residual organic matter, dead bacteria, and VBNC bacteria. Consequently, the correlation between RLU and CFU weakens, and ATP detection is not suitable as a surrogate indicator for evaluating disinfection efficacy. This finding is consistent with previous studies (Olafsdottir et al., 2018; Olafsdottir et al., 2017). Furthermore, Kwakman demonstrated that post-manual-cleaning ATP values cannot reliably predict microbial compliance after HLD (Kwakman et al., 2022).

Importantly, the clinical utility of ATP bioluminescence testing is highly dependent on the chosen threshold and detection system. In this study, we adopted a threshold of ≤45 RLU based on the manufacturer’s recommendations for the Ruhof ATP Complete^®^ system. However, this threshold has not been calibrated against the international standard ISO 15883-5, which defines an action level of ≥22 femtomoles of ATP per square centimeter. The lack of standardized conversion coefficients between RLU, a relative and device-specific unit, and absolute ATP concentration expressed in femtomoles per square centimeter means that our RLU cutoff cannot be directly translated to other ATP detectors. Different detectors may have varying sensitivities, reagent formulations, or luminometer designs. Consequently, our finding that ATP testing can serve as a useful screening tool applies strictly to the specific equipment, threshold, sampling method limited to the biopsy channel, and procedural workflow described herein. For broader implementation, for instance as a general quality control tool across different endoscopy units or countries, future studies should align their RLU thresholds with ISO 15883-5 by establishing conversion factors or using devices that directly output standardized units. Until such calibration is performed, comparisons of ATP results across different systems remain problematic, and caution is warranted when generalizing our conclusions to other settings.

## 5 Limitations

First, this study was limited to colonoscopes and exclusively to the biopsy channel; findings may not generalize to other types of endoscopes, such as duodenoscopes, or to other sites of the colonoscope, such as the external surface or the air/water channel. Second, we did not assess protein residues, nor did we include blank or negative controls. Third, bowel preparation quality was not systematically recorded. Although we excluded therapeutic procedures, and baseline ATP values did not differ significantly across groups (*P* = 0.305), residual confounding due to variations in bowel cleanliness cannot be entirely ruled out. Fourth, the ATP threshold used in this study (≤45 RLU) was manufacturer-defined and not aligned with ISO 15883-5, limiting the direct generalizability of our ATP-related conclusions to other detection systems or uncalibrated thresholds. Fifth, all bedside precleaning procedures were performed by a single dedicated nurse. While this reduced operator-related variability and strengthened internal validity, it also limits external validity to clinical settings with different personnel. The inability to blind the precleaning operator is inherent to this type of intervention, but outcome assessors were blinded to mitigate detection bias. Future multicenter studies involving more operators, broader sampling, and standardized ATP units are needed to validate and extend our findings.

## 6 Conclusion

Through systematic evaluation of multiple factors and their interactions on colonoscope reprocessing efficacy, combined with range analysis, ANOVA, and cost-benefit assessment, this study identifies an optimized colonoscope precleaning protocol for biopsy channel decontamination: alkaline detergent as the precleaning detergent, 4-hour solution replacement intervals, 20-second suction duration, and a clinically adaptable delay time not exceeding 3 hours. Furthermore, the research confirms that ATP bioluminescence testing can serve as a useful screening tool for biopsy channel cleaning quality.

## Data Availability

The raw data supporting the conclusions of this article will be made available by the authors, without undue reservation.
